# Supplementary figures and images for: Effect of sodium (S)-2-hydroxyglutarate in male, and succinic acid in female Wistar rats against renal ischemia-reperfusion injury, suggesting a role of the HIF-1 pathway
Source: PeerJ. 2020 Jul 10;8:e9438. doi: 10.7717/peerj.9438 (PMC7357568; doi:10.7717/peerj.9438)

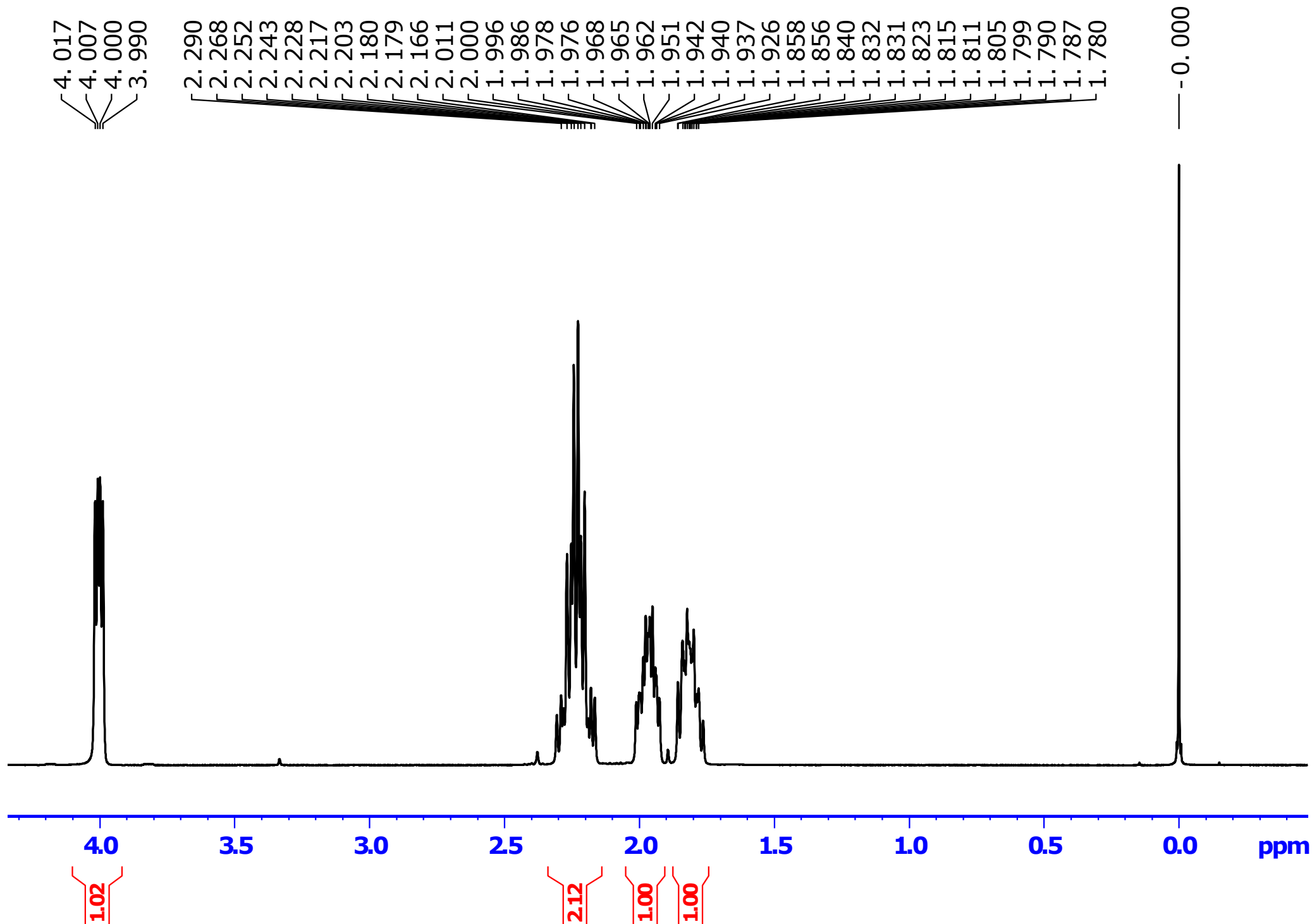

Supplement: Supplemental Information 3 [file peerj-08-9438-s003.pdf]

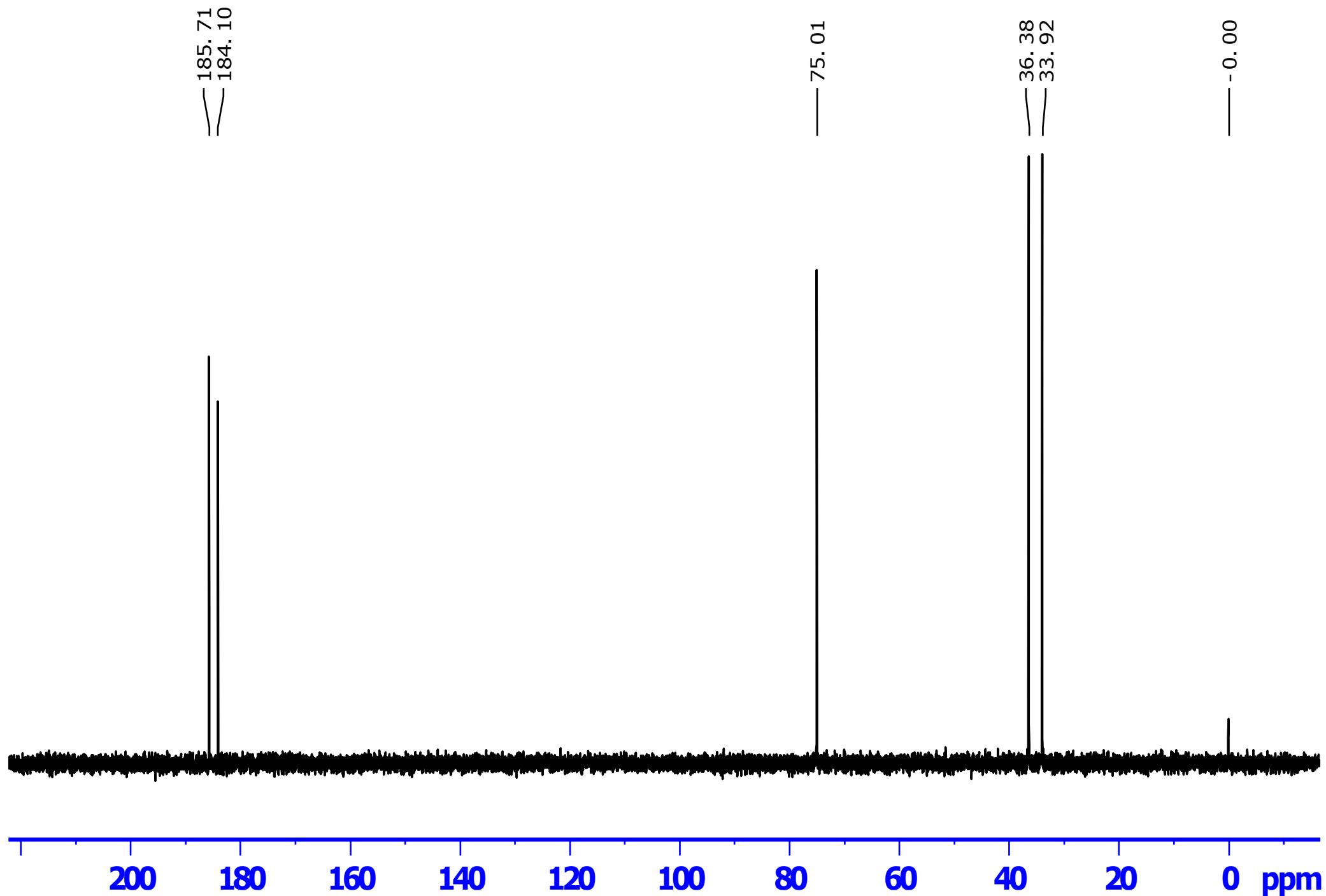

Supplement: Supplemental Information 4 [file peerj-08-9438-s004.pdf]

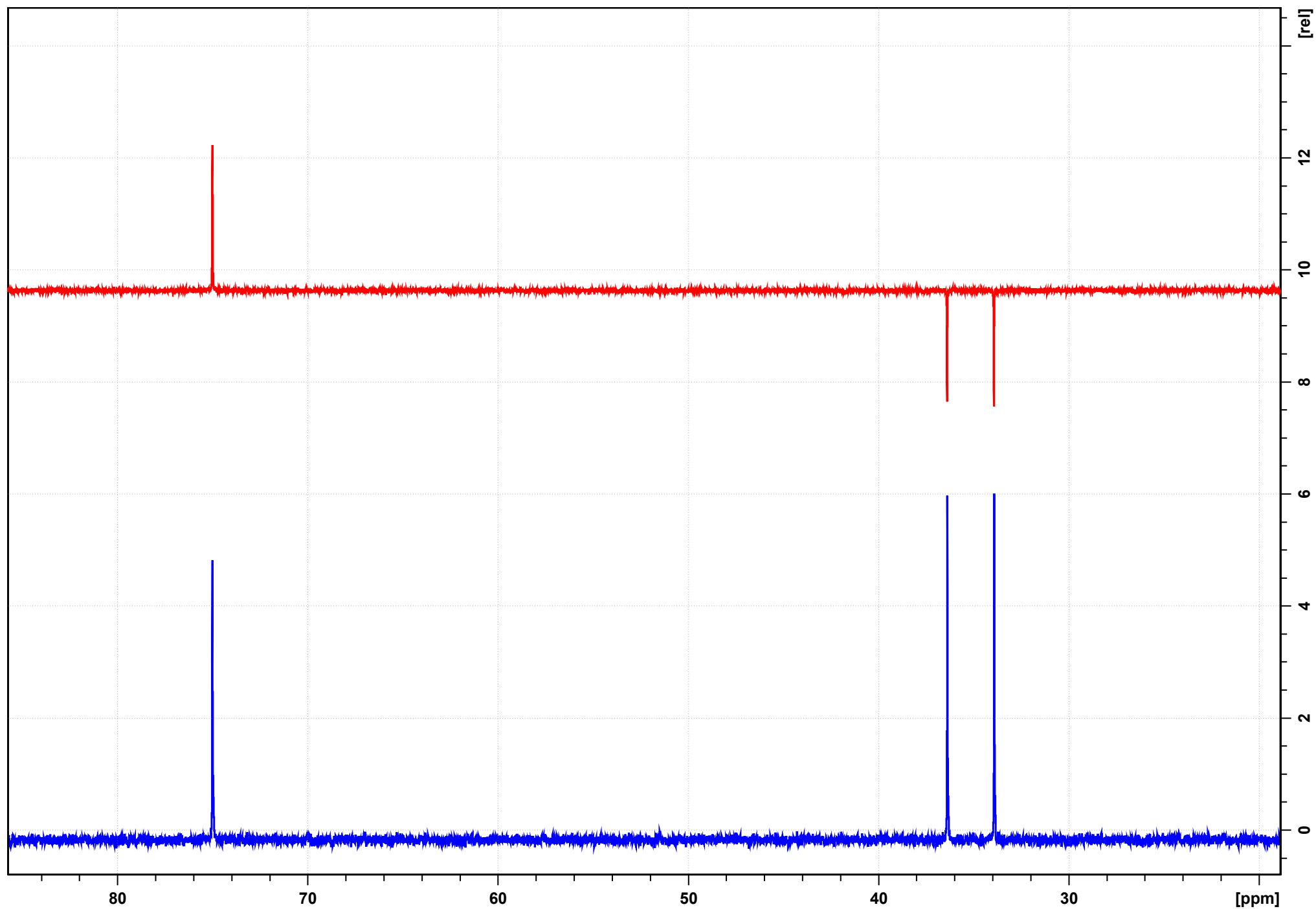

Supplement: Supplemental Information 5 [file peerj-08-9438-s005.pdf]

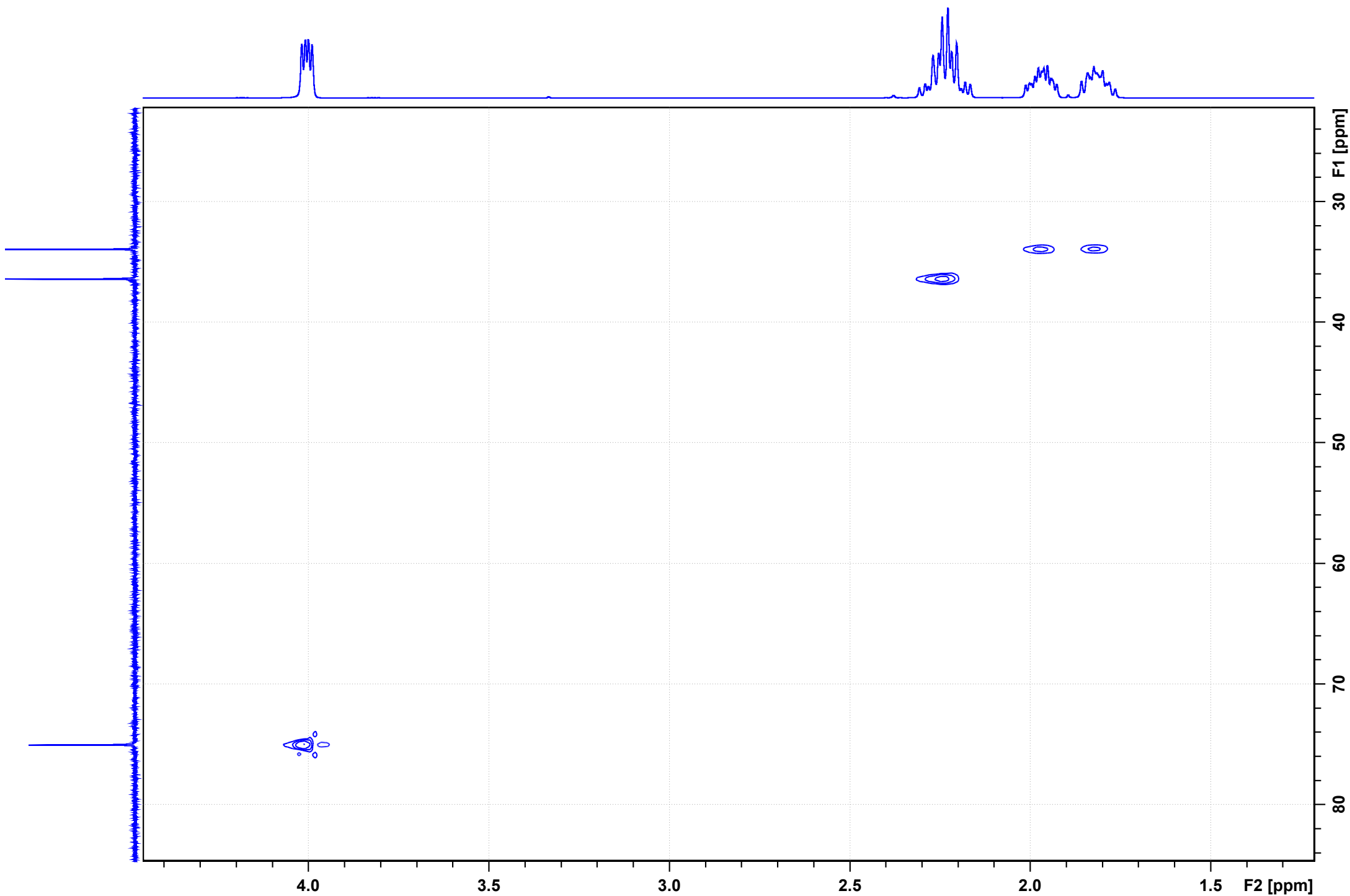

Supplement: Supplemental Information 6 [file peerj-08-9438-s006.pdf]

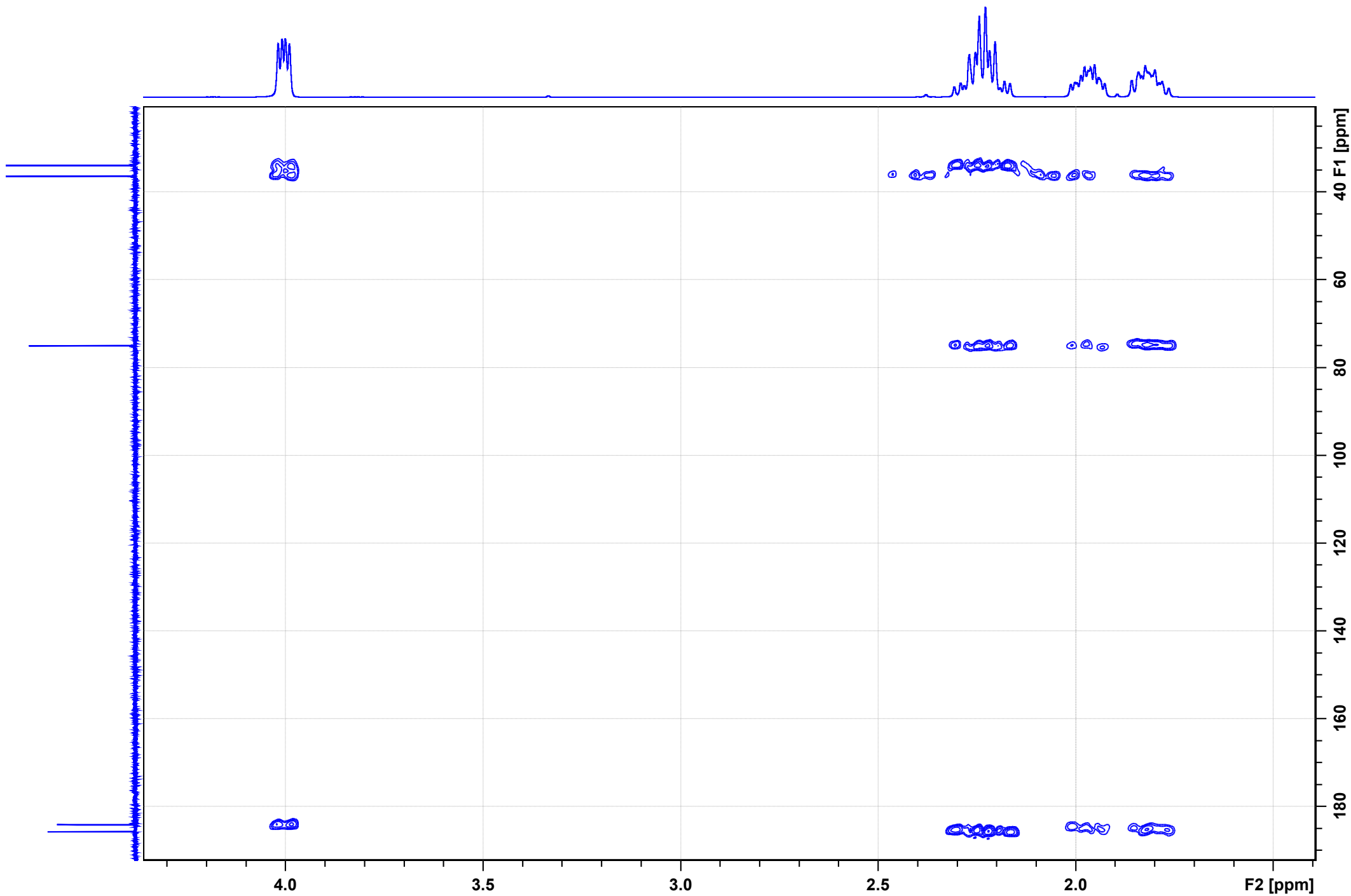

Supplement: Supplemental Information 7 [file peerj-08-9438-s007.pdf]
